# Interactive Patient Safety and Quality Improvement capstone during transition-to-residency program: virtual and in-person focused workshop for EPA 13

**DOI:** 10.1186/s12909-023-04220-5

**Published:** 2023-04-12

**Authors:** Catherine Chen, Kristen M. Coppola, Paul Weber, Payal Parikh

**Affiliations:** 1 Department of Medicine, Rutgers University Robert Wood Johnson Medical School, Director of Internal Medicine Bootcamp Program,, 125 Paterson St., Suite 2300, New Brunswick, NJ 08901 USA; 2 Department of Psychiatry, Rutgers University Robert Wood Johnson Medical School, New Brunswick, New Jersey USA; 3 Department of Medicine, Rutgers University Robert Wood Johnson Medical School, Associate Dean of Continuing Medical Education, Thread Director, Health Systems Science Curriculum, New Brunswick, New Jersey USA; 4 Department of Medicine, Rutgers University Robert Wood Johnson Medical School, Vice Chair of Quality and Safety, New Brunswick, New Jersey USA

**Keywords:** Communication, Medical education, Patient safety, Quality improvement, Transition to residency, Team-based learning

## Abstract

Identifying systems failures and contributing to a safety culture is the Association of American Colleges (AAMC’s) thirteenth Entrustable Professional Activity (EPA). While most curricula teach Patient Safety (PS) and Quality Improvement (QI) principles, student participation in live QI/PS activities remains limited. This workshop enabled late Clerkship phase students to apply these Health Systems Science (HSS) principles to real adverse patient event cases through team-based simulation.

This 3-h capstone included both a didactic review of QI, PS, and TeamSTEPPS® tools and an experiential component where student-led interactive small group discussions were augmented by resident and faculty preceptors. Collaboratively, students composed an adverse patient event report, conducted a Root Cause Analysis (RCA) during role-play, and proposed error prevention ideas after identifying systems problems. In April 2020, the in-person workshop became fully virtual due to the COVID-19 pandemic.

A statistically significant increase in ability to identify Serious Safety Events, Escalation Chain of Command, and define a Plan-Do-Study-Act (PDSA) cycle was observed. Comfort with RCA increased from 48 to 87% and comfort with TeamSTEPPS® principles increased from 68% to 85.5%

This novel capstone provided students with the tools to synthesize HSS concepts through problem-solving processes and recognize EPA 13’s importance. Their increased capability to identify appropriate chain of command, escalate concerns, and recognize serious adverse patient events also has training and practice readiness implications.

## Introduction

The AAMC’s thirteenth Entrustable Professional Activity (EPA) outlines expectations for medical students to identify systems failure and contribute to a culture of safety [1]. While most Health Systems Science (HSS) curricula teach principles of Patient Safety (PS) and Quality Improvement (QI), opportunities to actively participate in QI/PS discussions are not uniformly available to all students during their clinical years [2]. Program directors rated the proficiency of residents on the EPAs and felt a significant portion were under-prepared for EPAs 4 (Orders & Prescriptions), 7 (Evidence Based Medicine), 8 (Transitions of Care), 11 (Informed Consent), and 13 (Culture of Safety) [3].

As part of our four-year HSS Curriculum Thread, students partake in activities that expose them to QI/PS concepts through multiple modalities. The curriculum includes the evidence based system of healthcare improvement provided by the Agency for Healthcare Research and Quality (AHRQ) via TeamSTEPPS® (Team Strategies and Tools to Enhance Performance and Patient Safety) [4], online QI/PS modules provided by the Institute for Healthcare Improvement (IHI) [5] and American Medical Association (AMA), and the integrated High Reliability Organization (HRO) principles [6] used by our partner health care system.

Within the first several months in the matriculation, students begin their QI/PS journey. Students also are required to complete online QI/PS modules from the IHI and attend a didactic lecture on HRO principles. Subsequent activities are based on the flipped classroom model. Exposure to multidisciplinary PS huddles at our primary teaching hospital establish foundational understanding of QI/PS early in the medical school curriculum.

Despite longitudinal exposure to TeamSTEPPS® and QI/PS concepts through pre-clerkship years, we uncovered a deficit in active exposure to QI/PS after the core clerkships are completed. We implemented a HSS capstone during their required Transition to Residency (TTR) bootcamp which is given during the spring right before the students’ graduation. While team-based learning through mannequin simulation during residency has been described by Lu et al., [7], the goal of this capstone was to reinforce QI/PS concepts in the late clerkship period. We aimed for students to apply learnings from the preceding four years to actual adverse patient event cases in a simulated environment. The cases were intended to highlight multidisciplinary collaboration and key communication or systems challenges in a simulated approach.

The COVID-19 pandemic forced us, along with 78% of medical schools to quickly adapt virtual teaching modalities [8] and continue education despite decreased staffing. Flexibility in faculty time demands is always valuable, especially as the COVID pandemic evolves. We instituted more peer-facilitation and used team-based learning principles [9] to lead students through practical applications of PS/QI concepts, the results of which are described here.

## Methods

The TTR program for 4^th^ year medical students is a mandatory specialty specific 2-week long culmination of the medical school curriculum. It is typically run during the first weeks of March and April. During this program, students focus on practicing a litany of skills from informed consent to medical decision making to order placement in preparation for residency. Likewise, the QI/PS workshop described here concludes the longitudinal curricular thread for HSS and systems-based practice.

Undergoing its own QI process, the workshop was delivered over three consecutive years from 2019 to 2021 with some modifications in didactics and design. The 3-h capstone includes a didactic review of QI, PS, and TeamSTEPPS® tools, and an experiential component where students practice these skills via simulation. The design of this workshop models the QI/PS process starting with discovery of the adverse patient event and mimicking what residents and physicians would encounter in the clinical environment.

The workshop begins with didactics from faculty experienced in QI/PS. The large group is then divided into teams of 7–8 students. Discussions are led by student facilitators using a student workshop guide and RCA worksheet. Within the small groups, the students first collaboratively compose an incident report for a featured case using the Situation, Background, Assessment, and Recommendation (SBAR) format. Once completed, there is active adverse patient event dissection with “interviewing” of the students acting as participants involved in the case, simulating an actual RCA conducted under the auspices of a Safety Committee. Through this activity, the small group produces an RCA via a Fishbone Diagram and identifies potential solutions using TeamSTEPPS® and HRO principles as members of a “Patient Safety Committee”. The small group discussion is intended to focus on systems issues and not individual culpability. The large group then reconvenes. Here, each small group reports out components of their findings on the RCA and the TeamSTEPPS® and HRO solutions through a simulated Safety Huddle. Communication strategies that promote a culture of quality and safety are reviewed and discussion is concluded by revealing the outcome of the case upon which the workshop was based.

The discussions are augmented by faculty and senior resident preceptors who are available to each of the small groups. They assist discussions on reporting patient safety events, chain of command, and classification of patient safety events [10] (near miss, precursor safety event, and serious safety events) using a faculty preceptor guide. Preceptors representing all specialties are available to allow for diverse discussion and to emphasize the importance of QI/PS in medicine regardless of specialty choice.

Due to the COVID pandemic, the April 2020 and both AY2021 student cohorts received an entirely remote workshop through virtual conferencing software (Zoom). The same materials were used in both the in-person and remote versions with minimal modifications made for workshop flow. At the end of the 3-h session, the students submit the completed worksheet electronically.

Pre and post-test assessments of knowledge outcomes have been measured since 2019. Questions included definitions of RCA, HSS, Plan-Do-Study-Act (PDSA), identification of handoff tools from TeamSTEPPS®, and classification of serious safety events. In 2020, the outcome measure of interest for pre and post-test assessment also included several application-based questions (i.e., what model best describes how the error happened in this case; what type of patient safety event occurred in this scenario; and when a serious safety event occurs, who should it be reported to?). Ratings of comfort and exposure to performing RCA, confidence in applying TeamSTEPPS® principles were assessed. Students also rated the effectiveness of the workshop in their learning about patient safety (1 = not very effective; 5 = extremely effective).

## Results

**Fig. 1:**
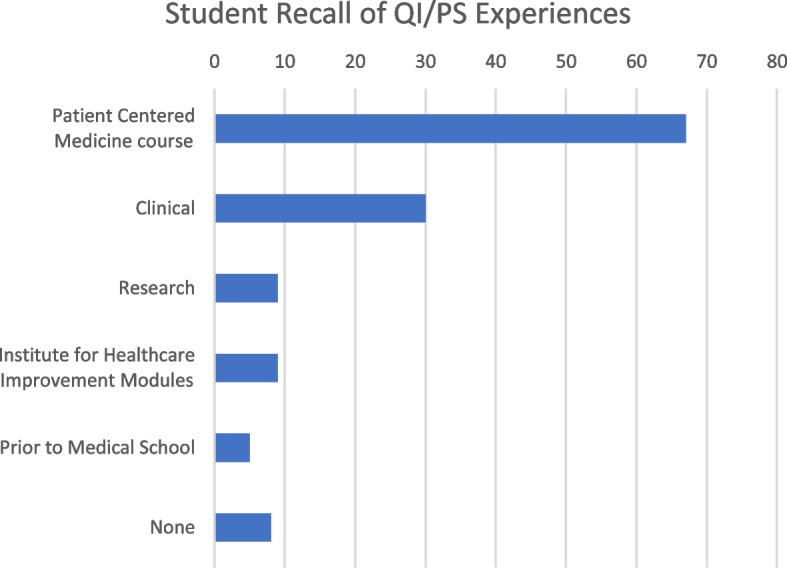
Number of students recalling Quality Improvement (QI)/Patient Safety (PS) experiences (*N* = 130); Patient Centered Medicine course (*n* = 67), Institute for Healthcare Improvement modules (*n* = 9), Clinical experiences (*n* = 30), Research (*n* = 9), Other (*n* = 5), None (*n* = 8)

One hundred and fifty-nine fourth year medical students who were in their final phase of training participated in the TTR courses from 7 specialty groups. We had a 98% response rate for the pre-test and 85% for the post-test. Prior to the workshop, 94.3% percent of students reported prior exposure to patient safety concepts during other courses at RWJMS. Most students (77%) recalled learning about these concepts didactically during the first- and second-year curriculum through our patient-centered medicine course and flipped classroom IHI modules; 31% also reported learning about the concepts during a clerkship or clinical experience (Fig. 1). In addition, 77.4% of students reported witnessing an adverse event during their clerkships. Despite strong didactic exposure and witnessing an adverse event, 78.6% did not have the opportunity to participate in an RCA.

Because of the extensive curriculum and students’ past-experience responses, we expected relatively high scores on items assessing their general knowledge about HSS. As predicted, 98% were able to correctly identify the definition of an RCA and there was no significant change at post-test. Seventy-eight percent of students correctly identified PDSA at pre-test which increased at post-test to 86% correct, but was not statistically significant (χ2 (1, *N* = 297) = 3.38, *p* = 0.066).

Process scores for individual items at pre and post-test can be seen in Table 1. Students correctly identified the model depicting how the error occurred 83% at pre-test and did not significantly change at post-test. For the other three process questions, what patient safety event occurred based on Safety Event Classification, who a serious safety event should be reported to (Chain of Command), and the primary tool used for transitions of care in TeamSTEPPS®, students scored significantly higher at post-test (χ2 (1, *N* = 297) = 9.67, *p* = 0.002; χ2 (1, *N* = 297) = 10.61, *p* = 0.001; and χ2 (1, *N* = 297) = 16.86, *p* < 0.001 respectively).

**Table 1:** Percentage scores for individual process knowledge questions at pre- and post-test

Question	Pre (*n* = 159)	Post (*n* = 138)	χ2	*p*
Error Model	83%	77%	1.24	0.265
Safety Event Classification	58%	75%	9.67	0.002
Root Cause Analysis	98%	96%	0.90	0.343
Plan-Do-Study-Act	78%	86%	3.38	0.066
Chain of Command	64%	81%	10.61	0.001
Transition of Care	84%	98%	16.86	< 0.001
Health Systems Science	66%	75%	3.08	0.792

Before participating in the workshop, 51% of student respondents reported that they were somewhat comfortable participating in an RCA and 2 students reported that they were extremely comfortable. Post-test, 59.4% were somewhat comfortable and 27.5% were extremely comfortable. Dichotomizing the variable into comfortable vs uncomfortable, 52% had some level of comfort at pre-test and 87.4% at post-test (χ2 (1, *N* = 297) = 70.12, *p* < 0.001). Similarly, at pre-test, 68.4% of students felt comfortable applying TeamSTEPPS® principles for communication and at post-test 88.5% reported feeling comfortable (χ2 (1, *N* = 297) = 21.78, *p* < 0.001).

In 2020, the workshop was successfully converted to a fully virtual format between the March and April bootcamp sessions. Evaluations from AY2020 showed that the workshop was rated extremely, very, or moderately effective by 80% of the March in-person students and 78.6% of April virtual students.

**Fig. 2:**
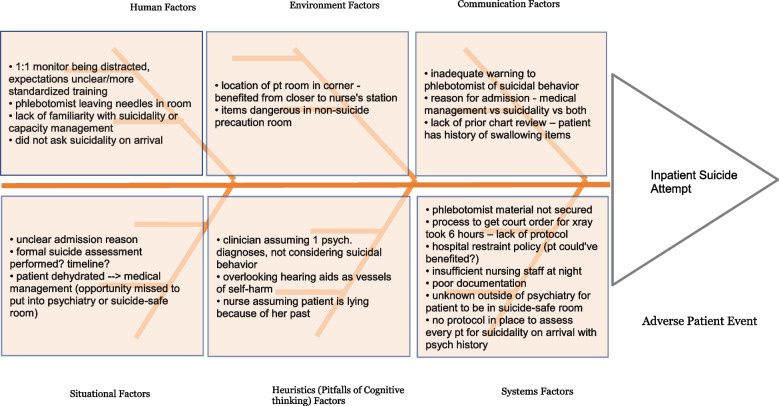
Sample Small Group Root Cause Analysis (RCA) Submission for a workshop case with inpatient suicide attempt where patient was placed under close observation with 1:1 but swallowed needles left by phlebotomy at bedside. Students generated this RCA based on a case and role-play interview

For AY2021, the entirely virtual workshop was well received and 93% of students reported that it was useful. Majority (47%) of open-ended student comments were focused on practical application of RCA, SBAR, and QI processes. Students also appreciated collaboration and different staff perspectives (28%). Fifteen percent of students also commented on the role-playing format as helpful to understand factors contributing to adverse patient outcomes. Sample comments and small group RCA submission are provided in Table 2 and Fig. 2.

**Table 2:** Sample Open-ended Comments

Collaboration
“Nice to work with people going into other specialties and to get their point of view as well”
“Just as in real life, working with multiple people to approach the root of the problem helps you overcome your biases/potential misses of root issues as well as increases the amount of ideas/solutions/recommendations”
“The case exercise was helpful in providing insight into the process from different points of view”
Process
“Very helpful to actually go through a root cause analysis. I've never done one before. “
“Walking through an example of Root Cause Analysis that outlined multiple factors in patient complication.”
“I also liked practicing with Situation, Background, Background, Recommendation (SBAR) as a communication tool.”
“The real-life fish-bone diagram exercise was very helpful.”
Case & format
“Having the students role play as members of the team was creative and more engaging
“Practicing learning the principles using a real case”

## Discussion

This capstone provides a novel way to help students synthesize HSS curricular concepts with real world patient cases. Having students work through the problem-solving processes of RCA can increase their appreciation for the importance of EPA13 and gain perspective on how QI/PS is operationalized in patient care scenarios. Beginning the workshop case from discovery of the adverse patient event mimics future residency adverse event experiences. Over the past three academic years, this workshop has evolved with increased emphasis on practical application. Students’ ability to identify appropriate chain of command, escalate concerns, and correctly recognize serious adverse patient events has implications in their post-graduate training and practice. Specific TeamSTEPPS® communication strategies give graduating students tangible and transportable skills to confidently contribute to a culture of quality and safety.

Qualitative reflections gave further insight into what students found most valuable from workshop participation. Practicing skills like SBAR or RCA with their group was most frequently noted with many also citing no opportunity to participate in PS events and RCAs before. Many students also mentioned that the case was thought provoking and they benefitted from examining the interdisciplinary causes and exploring “how many little factors can collide to create a very serious patient safety event.” These comments highlight students’ desire for more collaborative learning and interdisciplinary interactions. Qualitative reflections gave further insight into what students found most valuable from workshop participation. Practicing skills like SBAR or RCA with their group was most frequently noted with many also citing no opportunity to participate in PS events and RCAs before. Many students also mentioned that the case was thought provoking and they benefitted from examining the interdisciplinary causes and exploring “how many little factors can collide to create a very serious patient safety event.” These comments highlight students’ desire for more collaborative learning and interdisciplinary interactions. Based on this qualitative feedback, more opportunities may need to be created for student involvement in QI/PS efforts in clinical practice.

In this simulation model, albeit using real cases from faculty experience, generalizability to all patient safety situations may be limited. As this was the capstone to a longitudinal HSS curriculum thread, it is not intended to be used in isolation. We would recommend students have some basic working knowledge of QI/PS concepts, whether through modules or additional readings in preparation for this workshop.

Using student peer facilitators and resident preceptors increases engagement and decreases faculty supervisory burden. Minimal facilitator education is needed as the preceptor materials were designed to be self-explanatory. Though we did not study this directly, the resident as teacher opportunities model could also reinforce QI/PS concepts for resident preceptors. The plenary session incorporates residents and faculty from diverse specialties, encouraging students to recognize QI/PS as a component of every medical career. Preceptors were able to add nuance to discussions based on their own QI/PS experiences and viewed this session as a rewarding experience.

In 2020, the workshop was successfully converted to a fully virtual format without need for significant change to the distributed materials. Both in-person and remote students rated the course similarly. Small modifications in the workshop guides enabled interactive remote learning and affirmed the scalability of this exercise. Instructional format and facilitator flexibility has been *invaluable* as educational settings have evolved since the COVID-19 pandemic.

We began to pilot more case-based application questions in 2021 and future questions may include clinical reasoning components. As our HSS curriculum continues to progress, increased interprofessional collaboration can also increase simulation realism and applicability. In addition, we plan to conduct follow-up interviews with students to assess for lasting impacts to safety attitudes and changes in behaviors stimulated by this capstone.

## Conclusion

The transition to residency course provides a final opportunity especially for a formal HSS curriculum thread to reinforce QI/PS concepts for students at the cusp of graduation. This workshop demonstrates the potential and flexibility of team-based learning in virtual and in-person settings. Synchronous exposure to different specialties in conjunction with collaborative, student-led format were key elements that increased student engagement. Students gained foundational skills through this capstone and were able to appreciate the future clinical application potential of this knowledge into their post-graduate training.

## Data Availability

Data and workshop materials can be provided upon request.
